# Noise as Diagnostic Tool for Quality and Reliability of MEMS

**DOI:** 10.3390/s21041510

**Published:** 2021-02-22

**Authors:** Faisal Mohd-Yasin, David J. Nagel

**Affiliations:** 1 Queensland Micro- and Nanotechnology Centre, Griffith University, Nathan, QLD 4111, Australia; 2 Department of ECE, The George Washington University, Washington, DC 20052, USA; nagel@gwu.edu

**Keywords:** MEMS, noise, diagnostic, quality, reliability

## Abstract

This perspective explores future research approaches on the use of noise characteristics of microelectromechanical systems (MEMS) devices as a diagnostic tool to assess their quality and reliability. Such a technique has been applied to electronic devices. In comparison to these, however, MEMS have much more diverse materials, structures, and transduction mechanisms. Correspondingly, we must deal with various types of noise sources and a means to separate their contributions. In this paper, we first provide an overview of reliability and noise in MEMS and then suggest a framework to link noise data of specific devices to their quality or reliability. After this, we analyze 13 classes of MEMS and recommend four that are most amenable to this approach. Finally, we propose a noise measurement system to separate the contribution of electrical and mechanical noise sources. Through this perspective, our hope is for current and future designers of MEMS to see the potential benefits of noise in their devices.

## 1. Introduction

Microelectromechanical systems (MEMS) are on-chip integrations of microsensors and microelectronics. The former detects changes in the system’s environment by measuring mechanical, thermal, magnetic, chemical, electromagnetic, or other conditions or phenomena, while the latter processes this information for user applications. In the case of actuation, the onboard microelectronics or outside information could signal microactuators to react and create some form of changes to the environment. Due to low-cost, small footprint, good performance, and low-power, MEMS products have replaced bulky devices in various applications, namely consumer electronics, automotive, medical devices, telecommunications, etc. The market revenue for MEMS in 2019 was USD 11.5 billion, and this is expected to increase to USD 17.7 billion in 2025, with a compound annual growth of 7.4% [1].

Due to the widespread application of MEMS, the quality and reliability of these devices are of paramount importance. All MEMS companies have a large division of reliability testing to ensure that their devices enter the market at the highest quality. Correspondingly, the companies that design products utilizing MEMS devices paid close attention to the reliability of their systems for use by end-users [2]. However, after operating for some time, some MEMS will have decaying outputs and eventually cease to operate. When that happens, the current procedure is for the maintenance engineer to replace the devices or the whole module altogether. However, in critical systems, such as airbag trigger, tire pressure monitoring system, rate-responsive heart pacemakers, defibrillators, etc., the malfunction could mean a difference between life and death. As more MEMS devices are being integrated into those systems, a complementary method to detect their quality and reliability is warranted.

The purpose of this perspective is to explore a future research approach, that is, on the possibility that noise can be a useful indicator of quality and reliability in MEMS devices that undergo a large number of cycles. The same possibility also applies to nanoelectromechanical systems (NEMS). However, MEMS products are sold by the hundreds of millions each year, so we focus on them. This technique has been reported on various electronic devices. A key review paper on that topic was published by Vandamme in 1994 [3]. He compiled research works that have been written on the linkage between electrical noise sources to the quality or reliability of bipolar junction transistor (BJT), metal oxide semiconductor field-effect transistor (MOSFET), junction field-effect transistor (JFET) photodiode, Zener diode, interconnect (wire), compound semiconductor, thin films, etc. According to Google Scholar, this paper has been cited 600 times as of February 2021, clearly demonstrating its impact. Therefore, we conducted a comprehensive literature review to find out if a similar approach has been applied for any MEMS device. In addition to searching major databases by using the keywords “MEMS AND reliability AND noise”, we also went through all papers that cite the top publications in MEMS reliability [4,5,6,7,8], as well as MEMS noise [9,10,11,12,13,14]. Furthermore, we inspected patents and trade journals for possible contributions from industries. After conducting these laborious exercises, our finding is that such an approach or technique has never been attempted on MEMS. The impending question is, why? One key factor is that in comparison to electronic and semiconductor devices, MEMS have much more variety of materials, structures, and transduction mechanisms. Correspondingly, we must deal with various types of noise sources and the means to separate them. This separation is necessary if we were to perform accurate diagnostic for specific devices.

The rest of the paper is organized as follows: In the second section, we describe the motivations for assessing the quality and reliability of MEMS devices. For that purpose, we also explore the possibility of their degradation over time. The third section summarizes the diverse materials, structures, and transduction mechanisms are at the core of the operation of MEMS devices, for which we comment on their influences on the noise of those devices. In the fourth section, we provide an overview of the most common electrical and noise sources noises, show and explain the measured noise spectra of generic devices, and propose the framework to find the linkage between noise sources to the quality and reliability of MEMS devices. Then, in Section 5, we survey the various classes of MEMS, and we identify four types that are the most suitable candidates for our main purpose. In Section 6, we propose the noise measurement system with combined electrical- and optical-based detections to separate the contribution of electrical and mechanical noise sources. Finally, Section 7 concludes this perspective.

## 2. Quality and Reliability of MEMS

All engineering devices and systems vary to some extent in their quality after production and before use. After this, they are also subjected to performance degradation over time. The decline in performance and eventual failure of appliances and automobiles is a widely familiar example. Some systems fail early in their life, so-called “infant mortality”. Others work well for a long time and then start to degrade and fail. These two aspects determine the fundamental shape of the well-known “bathtub” curve [15], which shows the failure rate as a function of time.

**Figure 1:**
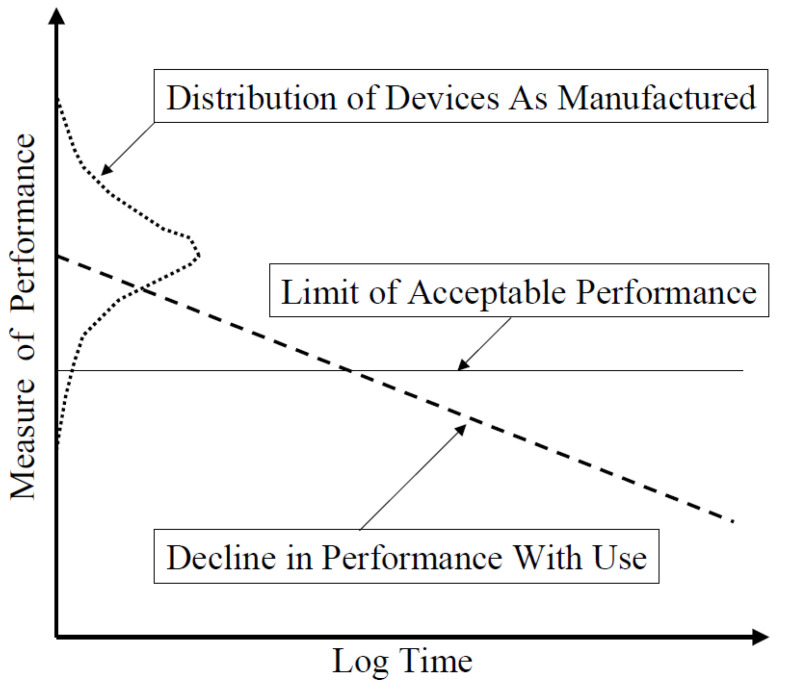
Schematic of the relationship between quality and reliability. The vertical axis represents some key performance parameters, such as the accuracy of a MEMS device. There is a distribution in such a parameter in the devices as they are manufactured and before their use (dotted line). Some devices have unsatisfactory performance; that is, they fall below the limit of acceptable performance (solid line), and therefore must be rejected. Devices above limit contributions to the yield of the manufacturing process. The dashed line shows a hypothetical linear decline in performance with time. Its intersection with the solid line gives the time at which a device in use no longer performs adequately.

The relationship between quality and reliability is illustrated in Figure 1. It indicates the “goodness” of a MEMS device, as quantified by the most important measure of its performance; both vary as-manufactured (quality) and degrades over time (reliability). It should be noted that the initial distribution in performance of devices is usually narrower than indicated in this schematic. Similarly, the rate of decline of performance in use is generally not very fast. Moreover, devices that begin in various parts of the initial distribution may have different rates of degradation. Despite these variations, the fundamental relationships as depicted in Figure 1 should hold.

Old and worn macroscopic systems become noisy because of the wear of rotary parts. The wear of industrial machines is of special concern. The use of vibration and noise measurements for such machines is commonplace. It is the basis of a practical discipline called “condition-based maintenance” (CBM). The central idea is to stop the operational use of a machine and service it only when its condition warrants the delay and expense [16]. This is a much more cost-effective approach than performing maintenance on a schedule, which can be wasteful. The goal of CBM is prognostic, i.e., the constant measurement of the noise in machines and the use of algorithms to predict when a system will need attention. Then, machine downtime and production scheduling are greatly improved. The same idea that is applied to ordinary-scale systems might prove useful for critical applications of MEMS devices, with one difference. MEMS devices are not subject to repair, so replacement is the only option if they ever degrade to a critical level.

Like its macroscopic counterparts, as MEMS devices age, they will exhibit increased levels of noise. When this happens, then it is possible to use noise measurements as indicators of the wear due to their use. This possibility is the micro-device equivalent of wear in ordinary macroscopic systems, such as knocking sound from the internal engine of old cars. It is noted that some MEMS, such as resonators and RF switches, go through billion of cycles during their lifetime [17]. That is, these MEMS and NEMS devices are far less susceptible to performance degradation than ordinary macroscopic engineering systems. Nevertheless, they can suffer changes in performance during repetitive use over many cycles. If these devices have digital output and thresholds have been set appropriately, the practical performance will not degrade with time and use. In contrast, those MEMS that operate in the analog domain can exhibit measurable decrements in their output after some operational time.

Another aspect of the utility of noise in MEMS should be mentioned herein. In most cases, the magnitude of the input signal must be well above the minimum detectable level for the production of a detectable output. However, in some cases, it is possible to get a measurable output from a device or system when the input is either noisy or pure noise. For the latter case, the noise signal itself excites some devices and systems to produce measurable signals or motions. This fact is exploited in the operation of some MEMS gas sensors [18].

## 3. Materials, Structures, and Mechanisms

MEMS devices for physical, chemical, or biological entities must have materials, structures, and mechanisms to do two things. The first function is to give a response to the quantity being measured (commonly known as the measurand). In the case of physical measurands, there is usually some deformation within the micromachined structure inside the MEMS sensors. In chemical and biological sensors, there is some molecular entity or other material built into the MEMS sensor to recognize the target molecules in the analyte. The second function is the transduction of the structural deflection or molecular recognition events into some signal that can be measured and recorded. These signals are subsequently converted into electrical quantities, such as voltage.

Due to their importance, the following subsections summarize different types of materials, structures, and transduction mechanisms that are used in making MEMS devices. Wherever appropriate, we comment on their influences on the noise characteristics of the devices.

### 3.1. Materials

MEMS fabrication processes grew out of the integrated circuit (IC) industry. However, the manufacturing of IC involves a much smaller set of materials and processes than the ones needed for MEMS devices. The most common materials in electronic chips include single crystal silicon and polysilicon, metals as interconnects (such as aluminum, copper, tungsten), and low dielectric constant materials as insulators. MEMS can contain any of these materials, plus piezoelectric, piezoresistive, optical, radio-frequency, magnetic, as well as chemically and biologically active materials. As a result, the noise behavior intrinsic to each specific material and the noise generation at interfaces between their many combinations must be considered. As MEMS devices are made of different materials, it is possible that noise from certain material(s) will be a much better indicators of their quality and reliability [19]. There is a related question of whether the different processing methods for the same materials within a MEMS device will result in different noise characteristics. Both intriguing questions are applicable for a wide variety of MEMS, and the combinations of materials and processes used [20].

### 3.2. Structures

IC are three-dimensional entities with complex internal structures. In contrast to MEMS devices, however, the structures within an IC are not made to move. There is a wide variety of types of moving structures within both developmental and commercial MEMS devices. If a structure can move, it can vibrate to accumulate cycles in either use or testing. Subsequently, the noise associated with mechanical motions is of interest. MEMS structures can be divided into several classes, which are listed herein. First, thin-film membranes supported on all sides by stiff frames are common MEMS structures, especially in pressure sensors [21]. Second, beams that are attached to solid support on both ends are normally found in radio-frequency devices, both as oscillators and switches [22]. Third, cantilevers that are attached at one end to their substrate are the basis of many chemical and biological MEMS sensors [23]. Fourth, flat surfaces that are attached to a frame by torsional hinges are the basis of many MEMS optical devices [24]. Fifth, interdigitated fingers, so-called “comb drives”, are found in MEMS accelerometers and other devices [25]. There are also other exotic structures in MEMS that do not belong to any of these classes.

### 3.3. Transduction Mechanisms

There are several main transduction mechanisms for MEMS devices [26]. In capacitive- and piezoelectric-based, MEMS sensors can employ deformations due to measurands to obtain output signals from either capacitor or from piezoelectric material, respectively. The reverse is true for MEMS actuators, i.e., the reciprocity principle applies. It should be noted that the noise characteristics for capacitive and piezoelectric devices are fundamentally different. There are a considerable number of publications on noise from the capacitor. The capacitor is important for traditional board-level circuitries [27], micron-scale domain, such as the most advanced switched-capacitor IC [28], and capacitive-based MEMS [10]. On the other hand, there is not much work being reported on noise from piezoelectric MEMS. Another mechanism for MEMS devices is based on the principle of piezoresistivity. It is widely employed in MEMS inertial sensors, such as accelerometers, pressure sensors, and microphones [29]. In such devices, small resistive elements are deposited on top of a thin membrane. When the membrane deflects in response to pressure changes or induced sound, the value of resistance changes, which is then converted to current or voltage; there are some studies of noise in resistive elements of the piezoresistive MEMS [30].

In addition to capacitive, piezoelectric, and piezoresistive transductions, there are other mechanisms that are employed in MEMS devices. Magnetic (or inductive), optical, and electron tunneling are examples [31]. However, due to the precision and small range of operations, we are in the opinion that the amplitude of the mechanical noise sources for these types of transductions is much smaller than piezoresistive, piezoelectric, and capacitive types. Moreover, the signal conditioning circuitries for these systems are more complex, as they need a sensitive amplifier, filter, and feedback mechanisms [32]. As a result, electronic noise is probably more dominant in these systems.

## 4. Noise Sources as Indication of Quality and Reliability

This section helps readers to understand the central idea that is being posed in this perspective. That is, can the noise characteristics of MEMS devices be indicative of their quality and reliability? Section 4.1 and Section 4.2 are intended to provide novice readers with the fundamentals of noise. The former covers the types and origin of electrical and mechanical noise sources, while the latter showcases and explains the generic noise spectra for most electronic or MEMS devices. We will also make a point on the need to separate the electrical and mechanical noise sources for our purpose. Finally, Section 4.3 discusses the type of quality and failure mechanisms that could be detected by specific noise sources. As shown in Table 1, such linkages for MEMS devices have not been established. Therefore, we propose two methods to collect this information from the vast literature. Those data are crucial to form the framework for the new approach.

### 4.1. Noise Sources

Noise in miniature electronic and mechanical devices has two basic fundamentals. The first is that the quanta (such as electrons, atoms, molecules, photons, etc.) that move inside those devices are discreet units. They are not a continuum of flows of matter and energy but rather the generation, loss, arrival and loss of discrete packets. The second is that many such quanta are involved in the operation of small devices. These large collections of individual quanta have statistical distributions that are fundamental to the appearance and character of noise. The first column of Figure 1 lists the major electrical and mechanical noise sources noise in MEMS. Next, we offer our explanation of the origins of these noise sources based on two previously described fundamentals. Noise is basically the uneven character of signals from a device or system, whether they are solely electrical or electro-mechanical. These signals are noisy. Since the particles are made of discrete quanta, they do not have a continuous flow. This forms the basis of shot noise [36]. Further, the quanta are moving within solids that have thermal vibrations called phonons. The thermally induced disturbance is known as thermal noise [33,34]. The interactions between carriers (such as electrons) and the phonons introduce further unevenness into the arrival rate of a current at the output of a device or system. In the case of semiconductors, the carriers (electrons and holes) can be generated by a variety of mechanisms, such as photon absorption and loss by electron–hole recombination. The rate of occurrence for these mechanisms is also random. When the carriers are lost, the phenomenon is called generation-recombination noise [40]. Another type of noise, i.e., flicker or 1/*f* noise [43], is due to the random trapping and release of carriers. They effectively appear or disappear during flows of currents and other processes. Both generation-recombination and flicker noises are related in that the carriers appear and disappear during the course of the operation of a device or system.

Mechanical noise sources have a lot in common with electrical noise sources. For the latter, electron or hole carriers are the sources of the noise, while for the former, atoms and molecules play an analogous central role. Thermomechanical noise [9] arises due to the presence of phonons within a small solid structure. The amount of energy being carried in different directions by the phonons is nonuniform. Hence, their impact with surfaces is not uniform in space or time, and that causes vibrations of the micro- or nanoscale structures containing the phonons. Brownian motion [49] is due to similarly random and uneven impacts of particles on the outside surface of a small particle or structure. Hence, thermomechanical noise and Brownian motion are conceptually related. The former is due to surface interactions from the inside of a particle or structure, while the latter is due to surface interactions from the outside of a particle or surface. Adsorption–desorption noise [54] is due to exterior atoms or molecules randomly sticking onto and escaping from the surface of a small object. It is also conceptually related to Brownian motion. Both involve exterior atoms or molecules. However, in Brownian motion, the incident quanta immediately bounce off of the target. In the case of adsorption–desorption noise, there is some residence time.

It should be noted that mechanical noise relevant to MEMS arises because of the very small size of the structures within such devices. Hence, the effects of phonons from within or atoms and molecules from outside the small structures can cause uneven (noisy) motions of the structures. Because the motions of the small component structures are central to determining the outputs of such devices, their signals are noisy due to such very small mechanical interactions. In the case of macroscopic structures, the effects of internal phonons and exterior atoms or molecules cannot be discerned due to a large number of such interactions and the inertial of the structures. However, large-scale particles, such as raindrops or hailstones, can cause macroscopic motions of correspondingly larger structures.

In Table 1, all electrical and mechanical noise sources within MEMS are independent of frequency, so their spectrum is flat. The exception is flicker or 1/*f* noise, which varies inversely with frequency. Flicker noise occurs in systems that are widely different in character and scale [52]. It is seen in large systems, such as the occurrence of slippage of grains in piles of sand, as well as the avalanches in the mountains. Fundamentally, it reflects the fact that very small events occur often; that is, they have a high relatively high-frequency and a correspondingly small 1/*f* value. In contrast, large events seldom occur, so they have a lower frequency and higher 1/*f* value.

### 4.2. Noise Characteristics of Electronic and MEMS Devices

**Figure 2:**
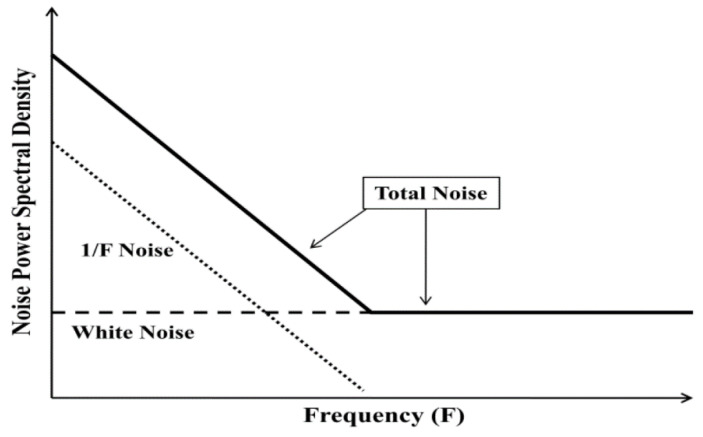
Schematic power spectral density (PSD) showing two types of noise as a function of frequency, white noise that is the same at all frequencies and 1/*f* noise that is important only at low frequencies. Both axes in this schematic are linear. However, most noise data are presented on log PSD vs. linear or log F graphs because of the wide variations in the noise intensity. Most experimental noise spectra are not as simple, but they often show these general characteristics.

Figure 2 is a sketch of the noise spectra observed in many electronic and MEMS devices, including our own results from measuring commercial MEMS accelerometers [57,58,59,60]. At low frequencies, the 1/*f* noise or flicker noise is dominant. Then, at a higher frequency, it decreases below the level of white noise. The schematic in Figure 2 indicates that there are four parameters that can be obtained from the measured noise spectra. The first and second parameters are the magnitude of the 1/*f* noise and the white noise, respectively. The slope of the 1/*f* noise, that is, any deviation from a simple inverse relationship, is the third parameter. Within the noise community, it is known as “alpha”. The fourth parameter is known as the “corner frequency”, in which the 1/*f* noise intercepts the white noise. The most important piece of information is actually the one that could not be seen from Figure 2. That is, the plot represents the combined noise spectra from both electrical- and mechanical noise sources, and we could not differentiate both contributions from that figure. In order to implement this approach for MEMS devices, this separation is necessary. Otherwise, we could not perform accurate diagnostics on the quality and reliability of specific MEMS devices. The proposed solution is presented in Section 6.

### 4.3. The Need for New Framework

In order for this approach to be applied to MEMS devices, the linkage between specific noise sources and devices must be established. For electrical noise sources, Vandamme [3] collected the information for various electronic devices. The details are extracted and recorded in the last column of Table 1. Since most MEMS contain onboard electronics, these data would be useful for our purpose, provided that they are used in combination with the ones for microsensors. For mechanical noise sources, despite our best efforts in searching the literature, we could not find published work that makes a direct linkage between these noise sources to the quality and reliability of MEMS devices. However, these linkages could be found by different means. We propose two indirect paths herein.

The first path is implemented by starting with the equation for a specific noise source and take note of the parameters that affect the device’s performance, i.e., quality. For example, the thermo-mechanical noise source is due to the atomic and molecular vibrations, and the equation [9] is given as $F_{thermomechanical-noise}=\sqrt{4k_{B}TR_{m}}$, where k_B_ is the Boltzmann constant, *T* is the absolute temperature, and R_m_ is the motional resistance (sometimes is referred to as mechanical resistance). Kaajakari [61] proceeded to derive the rms noise as $\bar{x_{n}^{2}}=\frac{4k_{B}TR_{m}}{c^{2}\omega^{2}+{\left(k-m\omega^{2}\right)}^{2}}$, where *c*, *m*, and *ω* are the damping coefficient, proof mass, and resonant or natural frequency, respectively. From both equations, one could observe the influence of thermomechanical noise on the quality of the MEMS device by looking at its performances at different temperatures under the additional influence of its proof mass, damping coefficient, etc. After identifying the relevant parameters, one should collect and analyze publications of all MEMS devices that report the existence of thermomechanical noise in their prototypes. The best-case scenario is when the direct relation between performance and that noise source is documented. In most cases, however, only the relationship between one of the key parameters of thermomechanical noise and the device’s performance(s) is plotted. After collecting data from a sufficient number of publications, one should see a trend. In other words, the data should indicate the types of MEMS devices that are most affected by thermomechanical noise sources. Finally, the same method could be iterated for other types of mechanical and electrical noise sources.

The second path involves studying the publications that report failure on their MEMS devices. This can be used to find the relation between noise and reliability. In 2003, van Spengen [6] wrote a highly cited review paper on failure mechanisms in MEMS. This is the best starting point, as subsequent publications that reported the failure of their MEMS devices would cite that review. The rest of the methodology is similar to what is proposed in the first path. One needs to read these papers, understand the cause of failure, and find information that links it to the specific type of noise sources that are reported in those failed devices. This “desktop study” could be performed without the need for laboratory equipment. However, it requires an enormous amount of time to sift through many papers. Furthermore, one needs an in-depth understanding of MEMS’s structure, fabrication processes, transduction mechanisms, noise mechanisms, and failure mechanisms. The result is a comprehensive framework that lists different types of electrical- and thermomechanical noise sources and the indication of quality and reliability of corresponding MEMS devices.

## 5. Choosing Suitable Devices-Under-Test

Which MEMS will be most amenable to this approach? To answer this important question, we have evaluated thirteen classes of MEMS for their value as devices-under-test. The classification is based on our 2010 review paper on noise in MEMS devices [11]. These MEMS devices are listed in the first column of Table 2. We include two types of inertial MEMS (accelerometer and angle rate sensor), three classes that respond to changes in pressure (pressure sensor, microphone, flow sensor), switches and mirrors for optical MEMS, switches and oscillators for radio frequency (RF) devices, three sensors used in liquids for flow, chemical and biological measurements, MEMS data storage devices, and magnetic MEMS. The second column shows the average operating bandwidth, and the third column shows the estimated maximum cycle. For the latter, we have taken the bandwidth and a constant operation time of one year (~3 × 10^7^ s) to estimate the maximum number of cycles during demanding applications.

The last column in Table 2 contains our opinion on the suitability of a specific class of MEMS for this new approach. We consider three aspects. The first is commercial maturity, as it is important to study the quality and reliability of devices that are used in very large numbers in critical applications. The second factor is the availability of noise studies for that device in literature. If there are considerable reports, we do not have to start from scratch on the theoretical analysis and experimental setup. The third factor is the complexity of testing. In order to design the setup, we must take into consideration the type of motion that needs to be supplied to test the devices. For example, a linear motion, as in the case of an accelerometer, is easier to trigger and control in comparison to rotary motion for the gyroscope. Additionally, the length of the test (maximum cycles) must be taken into consideration. Based on the data in the third column of Table 2, it is not clear that chemical and biological sensors will be good candidates as they undergo a relatively small number of cycles during their lifetime. The devices that work at GHz frequencies are also not attractive candidates, as they can accumulate as many as 10^16^ cycles during their lifetimes. These include RF oscillators, MEMS data storage devices, and magnetic memory devices. The motions of such devices are generally very small, or else they could not operate at those high frequencies. Therefore, the MEMS devices that operate in the mid-frequency group (values being in the range of 10^9^ to 10^12^) are the most suitable candidates.

After going through the selection criteria, four classes of MEMS emerge as suitable candidates:

- Accelerometers: They are sold in very large numbers for use in many different types of applications. They are made of silicon, have interdigitated comb structures that move linearly, and generally use capacitive readout;
- Microphones: They are also commercially important. They involve membranes made of various materials and generally use capacitive readout;
- Optical switches: These are also significant commercially in optical fiber networks, such as the Internet, and in display devices. They are made of different materials, are flat structures on torsional hinges, and are generally controlled electrostatically;
- RF switches: Such devices are being utilized for microwave devices. They involve a diversity of materials and structures and are generally actuated electrostatically.

## 6. Separating the Contribution of Electrical and Mechanical Noise Sources

**Figure 3:**
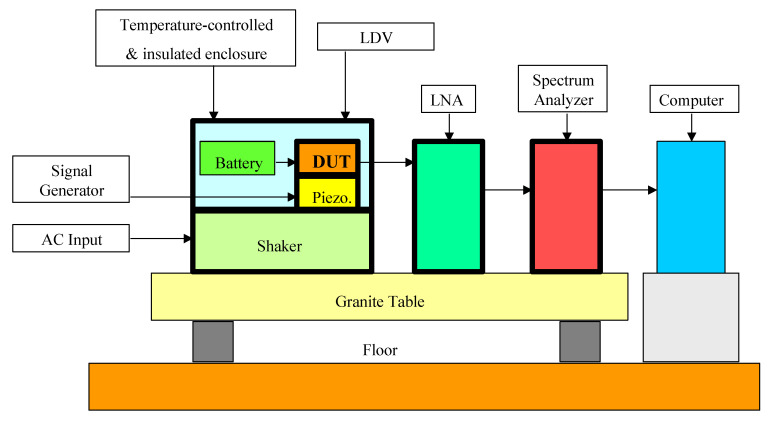
Schematic of the noise measurement setup. A granite table being suspended on air bearings will isolate the setup from external vibrations. The device under test (DUT) could be in open-die or package. A static measurement could be made by tilting the DUT with reference to gravity. The dynamic measurements could be made with the piezoelectric disc or shaker, each of, which has its own drivers, indicated to the left. In the electrical-based setup, the output signal from DUT is fed to a low noise amplifier (LNA) to provide signal gain. Then, the amplified signal is sent to a spectrum analyzer to display the noise characteristics. In the optical setup, the laser Doppler vibrometer (LDV) is used to optically assess the motion of the microsensor on the open die. The data from both the spectrum analyzer and LDV will be stored in a computer. The separation of electrical- and mechanical-based noise sources is performed by signal processing software when the purely mechanical noise spectra from LDV measurement are deducted from the combined noise spectra of the spectrum analyzer. It should be noted that several components are not illustrated in Figure 3, including a speaker to introduce acoustic excitation and the means for heating or cooling the DUT.

As explained in Section 4.2, in order for this approach to be effectively applied on MEMS devices, there is a critical need to separate the contribution of electrical and mechanical noise sources. The proposed setup is shown in Figure 3. It is the modified version of the pure electrical-based system that we previously employed to measure the noise of commercial MEMS [37]. The basic modules from that system are explained as follows: The noise signal from DUT is sent to the low noise amplifier (LNA) for amplification. The amplified signal is then fed to the spectrum analyzer for displaying the graphical output. Its characteristics will be similar to the one presented in Figure 2. It worth repeating that this output plot contains a combination of the electrical- and mechanical noise sources from the DUT. Finally, the raw data from the spectrum analyzer will be stored in the computer for storage and processing. In the proposed setup of Figure 3, we have added the optical-based system, that is, the laser Doppler vibrometer (LDV), to assess the motion of the MEMS structure [75]. Such a technique needs to be applied to open-die structures. The differential laser beams from LDV are commonly used to detect the small mechanical vibrations of various MEMS structures. In our case, they are employed to detect the unforced vibrations of pure mechanical noise sources coming from the DUT. The LDV will store this information on its own computer. The final stage is to perform the separation, which is performed by signal processing. With the raw data that is obtained from LDV and spectrum analyzer, the pure mechanical noise spectra from LDV measurement are deducted from combined noise spectra of the spectrum analyzer to produce the electrical noise spectra.

The noise measurement setup must be as simple as possible for an important reason. We only want to measure the intrinsic noise from DUT and preclude external noise sources. The latter step is a very delicate process and has grown into a solid research field called “noise reduction techniques” [76,77]. The main principle is to systematically eliminate the external noise sources from the environment [78] so that the system only outputs the intrinsic noise of the DUT. As the first step, a granite table being suspended on air bearings will isolate the setup from the floor’s vibrations. Then, important modules must be enclosed in a metal enclosure to avoid electromagnetic interference in our setup, which includes DUT, batteries, LNA, and cables. There are also necessary measures with grounding [79], as well as the use of low noise electronic components.

After ensuring a very clean low noise measurement system, the next step is to excite the DUT to produce an output signal with noise. The setup in Figure 3 is designed for commercial accelerometers, although it could be modified for other classes of MEMS. For that setup, three major types of excitations could be applied: mechanical, acoustic, and thermal. The mechanical stimulus could be offered by three separate means. The simplest is the static test, which is by varying the angle of the accelerometer’s plane relative to gravity. The DUT can also be subjected to the dynamic test, which involves the imposition of a sinusoidal-varying motion using some mechanical oscillator to excite the accelerometer’s comb-drive. The excitation could be performed over small amplitudes using a piezoelectric disc or over large amplitudes using a commercial shaker. Figure 3 shows the positions of both stimuli. Since the piezoelectric disc is only a few millimeters in diameter, it could be placed under the DUT and be driven by a signal generator in the sonic and ultrasonic frequency ranges. A commercial shaker could provide bigger vibrations at a lower frequency range of 10 Hz to 20 kHz. The third mean of the dynamic test is done acoustically. As opposed to piezo disc or shaker, this noncontact waves from a loudspeaker could provide high-frequency motion to the DUT. The acoustic waves will set the housing into motion with amplitudes that depend on the acoustical absorptivity of the housing and the way in which it is mounted, as well as the relative position of the acoustic source. This acoustic test will be most suitable for MEMS microphones. In addition to the mechanical-based test, the thermal-based test permits accelerated testing of DUT [80]. A commercial thermoelectric module could be placed under the DUT to provide low temperatures down to -40 °C, while a distributed thin film resistor could be used to heat the devices up to 150 °C. Finally, a thermocouple attached to the DUT will be used to measure its temperature. The thermal-based setup is not shown in Figure 3.

## 7. Conclusions

This perspective proposes a future research approach on the use of noise data as a diagnostic tool for assessing the quality and reliability of MEMS devices. We first explain the motivation for coming up with a complementary means of assessing the quality and reliability of devices that are being used in critical systems. Then, a comprehensive and thorough literature review was conducted. It was found that even though this technique is common for electronics devices, there is no evidence of a similar approach for MEMS devices. The answer is obvious. MEMS devices have far more diverse materials, structure, and transduction mechanisms. As a result, one must consider the electrical- and mechanical-based noise sources and a means to separate the contributions of both for this new approach to be employed effectively. We then listed the link between noise sources and the indication of quality and reliability of electronic devices. It was found through our literature search that such linkage for mechanical-based noise sources has not been reported, indicating a fertile research ground. Next, based on a set of criteria, we shortlist four classes of MEMS (accelerometer, microphone, RF and optical switches) as suitable candidates for the pilot study. After this, we propose a setup for performing the noise and reliability measurement. This system contains both electrical- and optical-based detection systems to be able to separate the contributions of electrical and mechanical noise sources from the devices-under-test.

Looking ahead, there is much work to be done. First and foremost, the framework that links electrical- and mechanical noise sources must be established. Second, from the description of MEMS’s fabrication processes, structure, and transduction mechanisms, it will be huge and challenging research to link between noise to quality and reliability on of MEMS devices, as they are many varieties, and one may be dominant over another. Third, the proposed setup could only separate the electrical and mechanical-based noise sources, while the identification of individual noise sources, such as thermal, 1/*f,* etc., remains a challenge. Nevertheless, the impact of solving these problems will be enormous, both intellectually as well as practically.

## Figures and Tables

**Table 1:** The name, mechanism, and indicative use of electrical and mechanical noise sources.

	Name	Mechanism	Possible Use to Indicate Quality or Reliability
Electrical noise	Thermal noise (also known as white noise, Nyquist noise [33], or Johnson noise [34])	Thermal vibration of the carriers when traveling across the conductor	For interconnects under strong bias, thermal noise measures the quality of heat contact. In addition, it also assesses delamination problems in metal-based films [35].
	Shot noise [36]	Random arrivals and departures of quanta	Shot noise indicates the quality of avalanche photodiode and Zener diode [37].
	Burst noise (also known as popcorn noise or random telegraph noise) [38]	Sudden step-like transition between two distinct steps	Burst noise is used as a quality indicator of BJT with crystal damage and quality of JFET and MOSFET in the sub-micron region [39].
	Generation-recombination noise [40] (also known as flicker noise)	Appearance and disappearance of carriers in semiconductors	Generation-recombination (G-R) noise indicates the quality of class III-V and II-IV compound semiconductors [41,42]. Unfortunately, its amplitude is superimposed by flicker noise in most cases.
	Flicker noise [43] (as known as 1/*f* noise or pink noise)	Trapping and release of carriers	1/*f* noise can be used as a complementary diagnostic tool for quality assessment of “almost all” semiconductor materials or devices. Vandamme [3] lists three factors that increase the 1/*f* noise, namely crystal defects being created by ions or protons, current constrictions and interfaces, and decreasing minority carrier lifetime. 1/*f^2^* is used to study the lifetime of various compounds [44,45,46]
Mechanical noise	Thermomechanical noise [9]	Effects of atomic and molecular vibrations on small structures	Although thermomechanical noise is widely published, such as [47] and [48], there is no known work that directly links it to the quality or reliability of a device.
	Brownian [49]	Random molecular impact of particles to the surface of the structure	Despite published papers, such as [50] and [51], there is no known work that links Brownian motion to quality or reliability.
	1/*f* mechanical noise [52]	Trapping and release of atoms/molecules	There is one work by Rocha et al. [53] that measured noise spectra of purely mechanical comb drive. The origin of 1/*f* noise is due to a defect that is thought to occur from the surface roughness of the moving structure.
	Adsorption–desorption noise [54]	Random residence times of molecules on surfaces	Despite published papers, such as [55,56], there is no known work that links adsorption–desorption noise to quality or reliability.

**Table 2:** Classes of MEMS devices, their bandwidth (BW), estimated number of cycles they experience during full-time use at maximum bandwidth for one year, and authors’ opinion on the suitability of each class for the proposed approach.

Class of MEMS	Bandwidth (Hz)	Maximum Cycles (log _10_)	Authors’ Opinion on the Suitability of the Device for the Proposed Approach Based on Three Criteria
Accelerometers	>5 K [62]	11	Mature products, a large number of noise studies, simple test setup using gravity
Angular rate sensors	<1 K [63]	10	Mature products, there are several noise studies, complex test setup due to rotary motion
Flow sensors	>100 [64]	9	There are some commercialized products, not many noise studies, complex test setup to precisely control gas flow or liquid flow
Pressure sensors	>5 K [65]	10	Mature products, not much noise studies, complex test setup to control the pressure using gasses
Microphones	>10 K [66]	11	Mature products, considerable database on noise studies, easy to test membrane deflection with speaker and controlled acoustical input
Optical MEMS-Switches	>1 K [67]	10	Mature product, not much noise studies, complex test setup
Optical MEMS-Mirrors	>100 K [68]	12	Mature products, not much noise studies, complex test setup
RF MEMS-Switches	>1 K [69]	10	Mature products, a considerable amount of noise studies, simple setup
RF MEMS-Oscillators	>1 G [70]	16	Mostly at the prototype level, a considerable amount of noise studies, long test times
Chemical sensors	~1 [71]	3	Mostly at the prototype level, there are some noise studies, tricky test setup as the output may degrade quickly
Bio sensors	~1 [72]	3	Mostly at the prototype level, there are some noise studies, tricky test setup as the output may degrade quickly
Data storage devices	~1 G [73]	16	Not many commercialized products despite being championed by IBM, not many noise studies, long test times
Magnetic device	~1 G [74]	16	Not many commercialized products, not many noise studies, long test times

## Data Availability

Not applicable.
